# A Multi-Stage Human Factors and Comfort Assessment of Instrumented Insoles Designed for Use in a Connected Health Infrastructure

**DOI:** 10.3390/jpm5040487

**Published:** 2015-12-16

**Authors:** Richard Harte, Leo R. Quinlan, Liam Glynn, Alejandro Rodriguez-Molinero, Thomas Scharf, Carlos Carenas, Elisenda Reixach, Joan Garcia, Jordi Carrabina, Gearóid ÓLaighin

**Affiliations:** 1 Electrical and Electronic Engineering, School of Engineering and Informatics, NUI Galway, University Road, Galway, Ireland; E-Mails: r.harte2@nuigalway.ie (R.H.); alejandro.rodriguez@nuigalway.ie (A.R.-M.); gearoid.olaighin@nuigalway.ie (G.ÓL.); 2 Physiology, School of Medicine, NUI Galway, University Road, Galway, Ireland; 3 CÚRAM Centre for Research in Medical Devices, NUI Galway, University Road, Galway, Ireland; 4 General Practice, School of Medicine, NUI Galway, University Road, Galway, Ireland; E-Mail: liam.glynn@nuigalway.ie; 5 Technical Research Centre for Dependency Care and Autonomous Living, Neàpolis Rambla de l’Exposició, 59-69, 08800 Vilanova i la Geltrú, Spain; 6 Irish Centre for Social Gerontology, Institute for Lifecourse and Society, NUI Galway, University Road, Galway, Ireland; E-Mail: thomas.scharf@nuigalway.ie; 7 CETEMMSA, Av. d’Ernest Lluch 36, 08302 Mataró, Spain; E-Mails: ccarenas@cetemmsa.com (C.C.); elisenda.reixach@eurecat.org (E.R.); 8 Cephis Wireless ULPA, Universitat Autònoma de Barcelona, Escola d’Enginyeria, 08193 Bellaterra, Spain; E-Mails: juan.garcia.paredes@uab.cat (J.G.); Jordi.Carrabina@uab.cat (J.C.)

**Keywords:** instrumented insole, gait analysis, comfort, human factors, human centered design, mHealth, eHealth, connected health, wearable electronics, older adult

## Abstract

Wearable electronics are gaining widespread use as enabling technologies, monitoring human physical activity and behavior as part of connected health infrastructures. Attention to human factors and comfort of these devices can greatly positively influence user experience, with a subsequently higher likelihood of user acceptance and lower levels of device rejection. Here, we employ a human factors and comfort assessment methodology grounded in the principles of human-centered design to influence and enhance the design of an instrumented insole. A use case was developed and interrogated by stakeholders, experts, and end users, capturing the context of use and user characteristics for the instrumented insole. This use case informed all stages of the design process through two full design cycles, leading to the development of an initial version 1 and a later version 2 prototype. Each version of the prototype was subjected to an expert human factors inspection and controlled comfort assessment using human volunteers. Structured feedback from the first cycle of testing was the driver of design changes implemented in the version 2 prototype. This prototype was found to have significantly improved human factors and comfort characteristics over the first version of the prototype. Expert inspection found that many of the original problems in the first prototype had been resolved in the second prototype. Furthermore, a comfort assessment of this prototype with a group of young healthy adults showed it to be indistinguishable from their normal footwear. This study demonstrates the power and effectiveness of human factors and comfort assessment methodologies in influencing and improving the design of wearable devices.

## 1. Introduction

Instrumented footwear can refer to any custom-made insole or foot-wear which incorporates electronic circuitry used to capture measurements such as physical activity, push-off, and contact forces, gait data, or health metrics [1,2]. An important consideration in the design of any kind of wearable device such as an instrumented insole is its comfort and human factors. Devices and systems used in healthcare settings, particularly those used in an unsupervised context, require high standards of comfort and human factors to facilitate technology acceptance, reduce gadget intolerance, and enhance the user experience, as well as to ensure the safety and comfort of the user. Recently, both the Food and Drug Administration (FDA) and the Agency for Healthcare Research and Quality have called for human factors evaluation of medical devices and systems during the design process [3,4] while knowledge of the user and their capabilities and characteristics has been highlighted as an important consideration within the design process [5].

It has been previously established that any improvement in subjective user scores on aspects of user experience of a device including ease of use, comfort, and cosmetic appearance, saw a proportional increase in the frequency of use of the prescribed device [6,7]. From these findings we can infer that comfort and human factors are key elements influencing the usability of prescribed footwear and that accurate comfort assessment is a critical activity within the design and testing process. Additionally, it has been established that the specific design, materials utilized and construction of footwear devices greatly influence their comfort [8].

Previous studies have sought to find objective and reliable measures of comfort for footwear and insoles, highlighting the subjective nature of comfort rating [9,10,11]. Recognizing these difficulties, we propose a systematic comfort and human factors assessment approach grounded in the principles of human-centered design [12] to influence the design of an instrumented insole. Our approach seeks to involve stakeholders, experts, and human users. This approach allows the application of simple, yet robust, comfort testing techniques within a cyclical process to mitigate possible problems before the device is exposed to potentially high-risk user groups, such as older adults with chronic conditions.

### The WIISEL System

**Figure 1:**
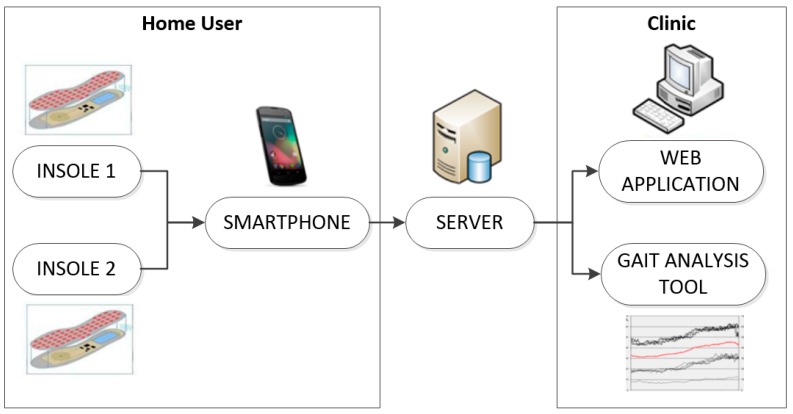
The WIISEL system architecture.

The Wireless Insole for Independent and Safe Elderly Living (WIISEL) system, developed as part of an FP7 project, is designed to continuously assess fall risk by measuring various gait and balance parameters associated with fall risk. The system is also designed to detect falls. The system consists of a pair of instrumented insoles and an associated smartphone which are both worn by the user. The insoles are inserted into the user’s shoes and worn on a continuous basis throughout the day. Data collected by embedded sensors in the insoles are sent to the smartphone, where they are then uploaded to a server in a clinic for processing and analysis. At this point the data can be presented in various ways to a specialist via a web app and desktop-based gait analysis tool. The overall architecture of the system is illustrated in Figure 1.

The system was designed to be worn by a user throughout their waking hours in order to identify specific gait patterns that may be contributing to a user’s fall risk. The system was targeted at older adults who represent a high-risk group for falls.

## 2. Methodology

Our methodology was designed using the principles of human-centered design with direct contributions from experts, stakeholders, and human volunteers. The process was designed to be iterative and consisted of three phases.

### 2.1. Process Phases

#### 2.1.1. Phase 1: Establish Context of Use and User Characteristics

In this phase a Use Case was constructed with input from all stakeholders. Constructing a use case is a commonly used method to capture user requirements and user preferences [13,14,15]. It normally consists of simple Universal Modeling Language (UML) diagrams. Our use case was an interactive, scenario and task-driven, descriptive document which provided a common platform for project stakeholders to communicate their vision for the insole’s role within the overall WIISEL system and the interactions it would have with the various system actors. The use case was used as a point of reference throughout this study and the wider WIISEL project to provide all stakeholders and analysts with the device’s context of use, and user characteristics.

#### 2.1.2. Phase 2: Expert Human Factors and Comfort Inspection of Prototype

Human factors inspection involves a multi-disciplinary expert group inspecting the device and attempting to identify human factors problems [16,17]. Human factors inspection is commonly used as a precursor to user testing. By identifying problems during the inspection process, it acts as a means to avoid subjecting users to testing devices which may be unsafe even for short periods of testing [18,19]. Our human factors inspection process consisted of individual experts inspecting the latest device prototype in a structured manner, attempting to identify potential comfort problems, using the use case as a reference.

#### 2.1.3. Phase 3: Human Volunteer Testing

**Figure 2:**
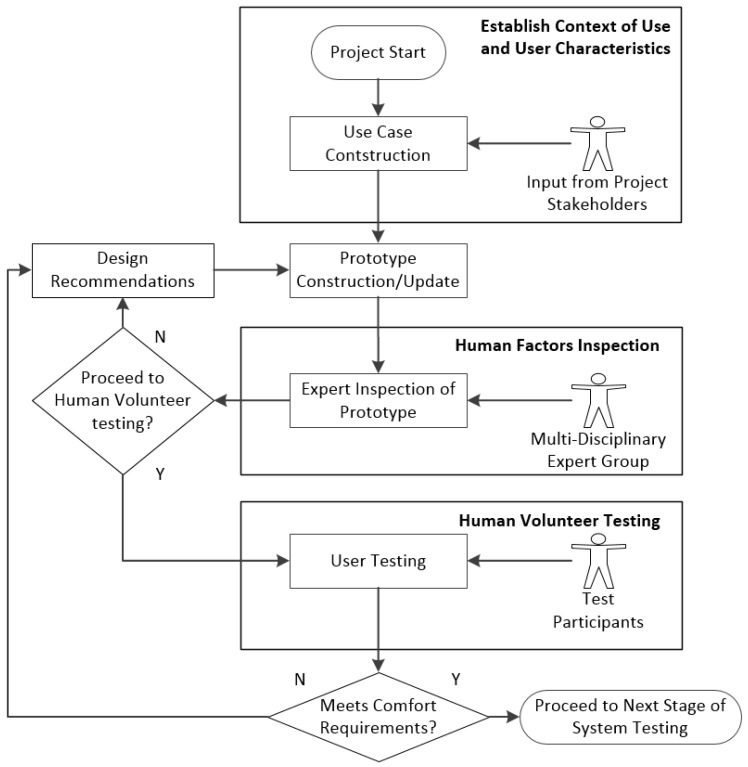
Iterative human factors and comfort assessment methodology.

Human testing involves monitoring volunteers while they wear the device and recording and documenting any problems the volunteer encounters [20,21]. Our human volunteer testing was informed by the recommendations from the experts during the inspections carried out in Phase 2. The full methodology utilized is illustrated in Figure 2. The methodology was designed to be cyclical, where decisions are made at different gating points of the process on whether to continue to the next phase of the process or to iterate the current phase. Most critically, expert consensus is used at the end of Phase 2 to decide whether or not a prototype is suitable to continue for human volunteer testing. If it is deemed not suitable for human volunteer testing, then suggestions are offered on how to improve the prototype to better meet comfort and human factors requirements. For human volunteer testing we sought to establish whether there was a statistically significance difference between a “normal” control footwear condition (the user wearing their typical footwear without the WIISEL insole) and the condition where the user was wearing the insole.

### 2.2. Use Case Development

**Figure 3:**
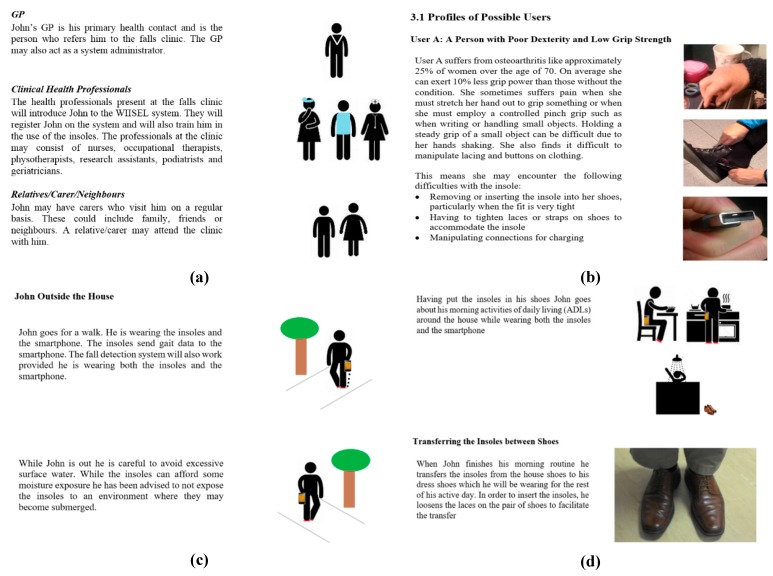
Screenshots of the WIISEL insole use case describing: (**a**) the different actors who would be interacting with the system; (**b**) the possible physical limitations of the target users; (**c**) how the user might interact with the insole in an outdoor setting; and (**d**) how the user might interact with the insole throughout the day.

The use case is a document designed to provide the context of use for the proposed device and to build a profile of a typical end user. The developed use case described in detail the scenarios in which the insole is prescribed and then used by an older adult in their daily life. The document was presented in a storyboard format and included illustrations, images, and functional information about how the user interacts with the insole (Figure 3). The use case was directly informed by contributions from the different stakeholders and was developed using an iterative process. In response to the completed and agreed use case, the first prototype insoles were built.

#### **Criteria Required to Proceed to Prototype Construction** 

All project stakeholders must agree that:

(a) The use case represented the correct context of use for the proposed the device/system.
(b) The use case presented an appropriate description of the target users of the system.

### 2.3. Prototype Construction and Electrical and Environmental Test

**Figure 4:**
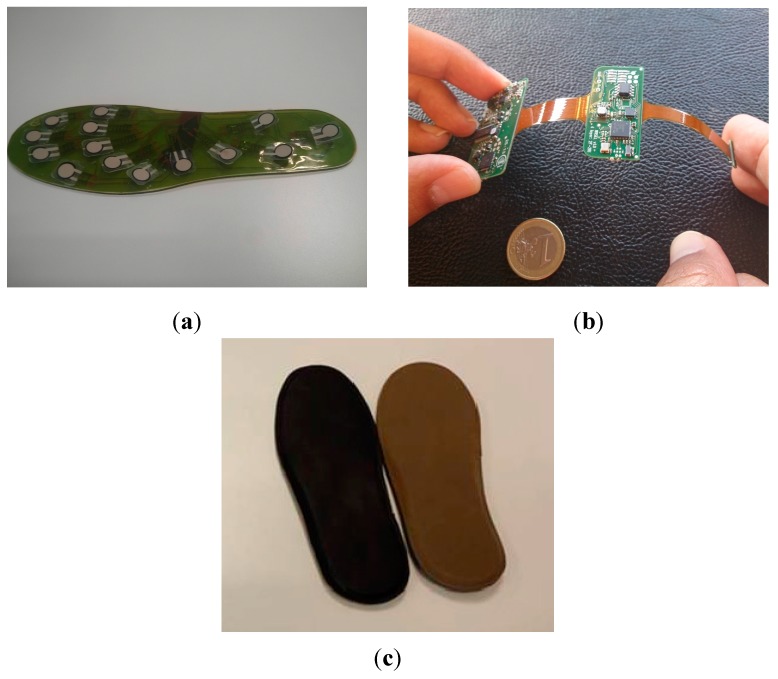
(**a**) 1mm polyurethane sensor layer; (**b**) PCB layer; and (**c**) top layer (black artificial leather) and bottom layer (Brown EVA Foam).

The WIISEL insoles consisted of a number of electronic components, which were encapsulated in the insole. Components included 14 pressure sensors, two inertial sensors, a microcontroller, a battery, an antenna and a charging coil. For the first prototype, the pressure sensors, integrated in a Kapton layer, were encapsulated by 1 mm of polyurethane (Figure 4a). This encapsulated Kapton layer was then connected to the PCB layer (Figure 4b) and these combined layers were then covered by a top leather layer and a bottom EVA foam layer (Figure 4c).

#### Criteria Required to Proceed to Human Factors and Comfort Inspection

Before being made available for human factors and usability testing, the prototypes were subject to numerous electrical and environmental assessments such as water ingress, electrical leakage/safety, sensor sensitivity and durability. Prototypes were only made available for usability and human factors analysis after designers were satisfied with their functional integrity.

### 2.4. Human Factors and Comfort Inspection of Prototype

A cognitive walkthrough protocol is a popular approach to human factors inspection and involves the inspectors carrying out an analysis of the device in the context of how the user would interact with it [22,23]. The inspection methodology was split into three parts:

(a) Establishing device characteristics, user profile, context of use through an analysis of the use case document
(b) Inspection of the device and problem identification
(c) Problem severity score and final recommendation

Six experts were recruited to carry out the inspection and all followed our defined methodology; their occupation and specialties are listed in Table 1.

**Table 1:** Description of each of the experts who were recruited to carry out human factors and comfort inspection of the insole.

Expert	Occupation	Relevant Expertise
1	Physiotherapist	Physiotherapist and clinical rehabilitation specialist at a primary care clinic
2	Professor of Podiatry	Professor of Podiatry and Head of the Discipline of Podiatry. This Expert has a specialist research interest in tissue viability and diabetic foot disease
3	Podiatry Researcher	Expert in Fall Risk and Diabetes in the Older Adult population
4	Clinical Podiatrist	Vast experience with biomechanical issues, orthotic prescription and insole design
5	Occupational Therapist	Experience working with community dwelling older adults and research interests in Fall Risk
6	Podiatry Researcher	Specialist in Foot Biomechanics and Arthritis

Human factors and comfort inspection sessions were carried out using a one-to-one format with the researcher. The expert was presented with the latest version of the WIISEL insole use case. Having established in what context the device would be used and who would be using it from the use case, the expert was formally asked whether they understood (a) the context in which the device would be used (context of use) and (b) the nature of the target end user (user characteristics). At this point the expert could ask for further clarification on the context of use or on the user characteristics, at which point the researcher who had an intimate knowledge of the system and the potential users would provide the required information.

The insoles were then shown to the experts and a cognitive walkthrough methodology [24] was employed to evaluate the quality, safety and ergonomics of the insoles. The expert studied the insoles in relation to the context of use described in the use case and was encouraged to think aloud and to explicitly identify problems as they examined the device [16]. Experts were made aware of how the insole was constructed and what materials were used. If any problems were identified by the expert, these were described in the expert’s own words. The full list of problems were then listed and read back to the expert by the researcher. The expert had an opportunity to then combine related problems into single problem statements or remove problems that they felt in retrospect were not critical enough to be listed. The expert then applied a severity score to each problem based on Nielsen ratings [21,24]. Severity scores ran on a scale of 0–4. Table 2 lists what each score means in usability terms and its potential implications for users.

**Table 2:** Severity Scores.

Score	Classified as	Implications for Future Design
0	Not a Usability Problem	Something to consider for future design iterations but will not affect general use
1	Cosmetic Problem	Need only be fixed if time, resources available. Problem should not affect the majority of users
2	Minor Problem	Low priority fix, problem will affect some users
3	Major Problem	Important to fix, high priority, fix as soon as possible, problem will affect majority of users
4	Catastrophic Problem	Must be fixed before product is tested with end users, problem will affect all users

All problems identified during the inspections were rank ordered in terms of how many experts identified the problem and the corresponding severity score. These two metrics were used to apply a severity rating to each problem identified. These, and other similar weighting systems, are commonly used to prioritize which problems are most important to fix [25]. The severity rating is defined as the number of times a problem was identified multiplied by the mean severity score. The maximum possible severity rating in this study for a particular identified problem was 24 (all experts identifying a problem and assigning it a severity score of 4). With this number, the problems could be prioritized and the priorities are defined in Table 3.

**Table 3:** Showing how total severity ratings are used to categorize problems based on priority.

Total Rating	Usability Implications
1–6	Cosmetic Problem; Should be fixed only if resources time are available, these problems should not affect the majority of users.
7–12	Low Priority Fix; Will cause problems for some users and should be addressed as soon as resources are available.
13–18	High Priority Fix; Will affect many users and lead to severe reduction in user acceptance, should be fixed as soon as possible.
19–24	Usability Catastrophe; Will affect all users and may cause danger, development should be halted until problem is fixed.

Finally the expert made a recommendation based on their inspection using a simple questionnaire, summarized in Table 4.

**Table 4:** Simple questionnaire for experts to provide recommendations for the user testing phase.

*This device is suitable for user testing with the following user groups* >>	Young Healthy Users	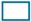	*And can be worn for a period of*	Please specify the maximum period of exposure you would recommend this device could be worn safely by each of the user groups:
	Healthy Older Adults with no Fall Risk	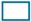		
	Older Adults with Fall Risk	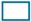		
	No User Groups	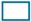		

#### **Criteria Required to Proceed to Human Volunteer Testing** 

Proceed to human volunteer testing if:

(a) There are no problems identified which receive a severity rating corresponding to a human factors catastrophe (See Table 3), or
(b) The majority of experts reached a consensus on whether older adults should be tested with the insoles or not. If the majority of experts (>3) selected, “No User Group” then it was deemed that the device must re-designed prior to human testing.

### 2.5. Human Volunteer Testing

In comfort assessment, it is considered best practice, to have a control condition or baseline measurement such that all experimental conditions assessed can be compared to the baseline measurement [10]. To provide a controlled observation of comfort of the prototype insoles, 10 participants were recruited for four sessions of comfort assessment over four consecutive days. The participants provided written informed consent and presented themselves at the Human Performance and Locomotion Laboratory in NUI Galway. Ethical approval for testing was granted by the NUI Galway Research Ethics Committee. Participants were screened to ensure exact fit with the insole and were excluded if they had a previous lower limb surgery, currently had an injury, or used an orthotic device. The itinerary for the four days of testing is outlined in Table 5. Before testing, participants were instructed to attend the lab in their most comfortable “typical daily walking” footwear (sandals, flip-flops, clogs, boots, heeled shoes, or open shoes were excluded) and to wear these for all four days of testing.

**Table 5:** Outline of activities carried out by users during user testing.

Day and Condition	Activity	Activity Time (h)
Day 1 (Normal Footwear without instrumented insoles)	Outdoor Walking	1
	Treadmill	2
Day 2 (Normal Footwear with instrumented insoles)	Outdoor Walking	1
Day 3 (Normal Footwear with instrumented insoles)	Outdoor Walking	1
	Treadmill	1
Day 4 (Normal Footwear with instrumented insoles)	Outdoor Walking	1
	Treadmill	2

**Figure 5:**
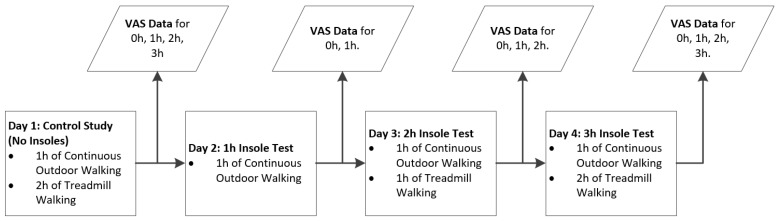
Procedure for testing insole comfort with young healthy volunteers.

Outdoor walking consisted of walking on paved roads around the city of Galway and around the NUI Galway campus. All participants walked the same route. Participants filled out visual analogue scales (VAS) at certain points during the testing. This is illustrated in Figure 5.

**Figure 6:**
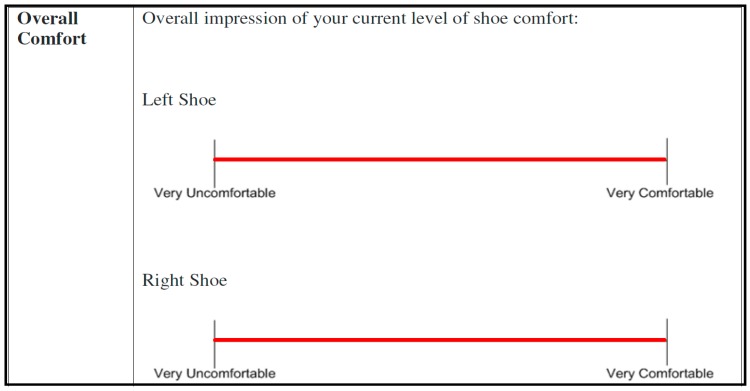
Example of the visual analogue scales used during user testing. Participants marked with an X or a vertical line along the scale where they perceived their current level of comfort.

VAS has been shown to be a reliable method for capturing personal comfort levels. Studies comparing the use of different types of VAS have shown that the sensitivity and reliability of VAS are somewhat influenced by the words used to anchor the endpoints, by the length of the VAS, and by other factors. Those VAS scores that most clearly delineate extremes (e.g., the best condition imaginable, the worst condition) and are 100–150 mm in length have been shown to have the greatest sensitivity and are the least vulnerable to distortions or biases in ratings [26]. Participants were all given the same written and verbal instructions about how to fill out an anchored continuous 100 mm visual analogue scale (VAS) with the left end labeled “Very Uncomfortable” and the right-side labeled “Very Comfortable” [10,11]. The questionnaire to be filled out at each stage of testing contained eight separate VAS querying the overall comfort of each foot as well as the comfort of specific part of each foot: the heel, midfoot, and forefoot areas of each insole. An example of the scales presented to participants is shown in Figure 6.

After each activity, the participants, as well as providing the VAS scores, also provided qualitative feedback on what aspects of the insoles they found comfortable or uncomfortable.

#### **Criteria Required to Pass Human Factors and Comfort Assessment** 

Comparison of mean VAS scores for all users between the baseline condition (normal footwear with no WIISEL insoles) and the insole condition (wearing the WIISEL insole in normal footwear) should show no statistically significant difference when comparing similar time periods of wear for each condition, if the insole is not affecting comfort. Statistical significance was demonstrated using paired *t-*tests (α = 0.05) [11].

## 3. Results of First Prototype (V1 Prototype) Test Cycle

The results of the human factors and comfort inspection and the subsequent human volunteer testing for the V1 prototype of the insoles are presented in this section.

### 3.1. Human Factors and Comfort Inspection

**Figure 7:**
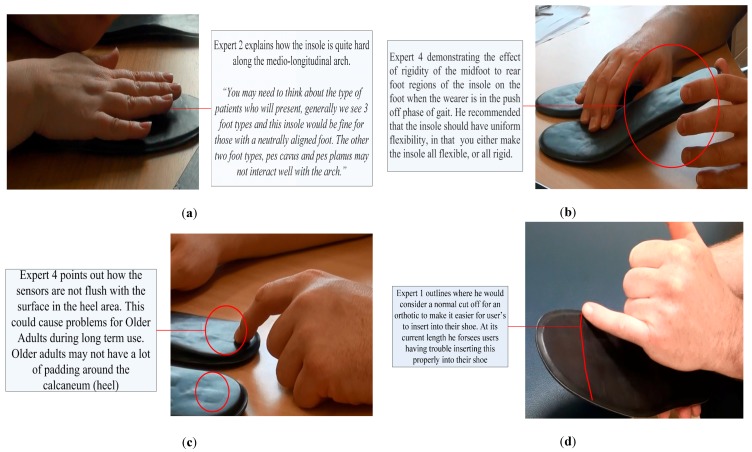
An expert (**a**) demonstrating how the firmness of the medio-longitudinal arch could cause discomfort for a user; (**b**) demonstrating the lack of flexibility in the heel-mid-foot region; (**c**) pointing to the protruding sensors at the heel of the insole; and (**d**) explaining how fitting problems might occur and where he would consider a normal cut-off for more universal fit insoles.

From reading the use case, all experts agreed that they understood the context of use and the user characteristics, which the device was designed for. The problems in order of priority based on the problem severity rating are presented in Table 6 and visual descriptions of selected problems are shown in Figure 7. The explicit description was based on the general consensus of the experts.

**Table 6:** Problems ordered in terms of a weighted aggregate of frequency reported and severity rating).

Problem Number	Problem Identified	Number of Experts Who Reported Same Problem (Range 1–6)	Severity Score Mean (Range 0–4)	Problem Severity Rating (Range 0–24)
1	The medial-longitudinal arch is too firm (the firmness of the insole in general was cited as a problem but the medio-longitudinal arch was cited as the most critical)	6	2.85	17
2	Lack of flexibility in the midfoot to rear foot region	4	3	12
3	Sensors are not flush with the surface of the insole	2	3.5	7
4	Length and thickness for manipulation and fitting	3	2.3	7
5	Pinch ridge around the outside of the insole causing problems for lateral movement and fit	3	2	6
6	Lack of a proper heel cup	2	2.5	5
7	Forefoot rigidity	1	3	3
8	Slippery surface	2	1.5	3

### 3.2. Expert Recommendations

All experts agreed that the insoles could be tested on young healthy users. Only half of the experts agreed that they could be tested on older adult users. One expert suggested that they could be tested on older adults with a high fall risk. These recommendations are summarized in Table 7.

**Table 7:** Expert recommendations for what groups can be exposed to user testing.

	Young Healthy User (under 60)	Healthy Older Adult User (over 60)	Older Adult User with High Fall Risk
Percentage of Experts who Approved Use with this Group	100%	50%	16%

All experts also agreed that the manner of exposure to the insoles should be extremely controlled with close observation. A common suggestion was to allow the user to wear the insole for one hour on the first day, and then add an hour of use for every day after until eventually the insole could be worn on a continuous basis after a period of 1–2 weeks. These points are reflected in a range of comments made by experts during the human factors and comfort inspection sessions:

“For any kind of device (referring to customized orthotic insoles) we would introduce to the shoe in the clinic, we would ask the patient to wear for 10 min to assess the comfort and then build up to full-time wear within two weeks. Typically, clinicians allow patients a break-in period of about two weeks during or after which orthotics/devices may be modified or withdrawn altogether.”

“When somebody puts something into their shoe, it’s going to take time to get used to it, so usually you break into it, maybe for an hour a day. There has to be a lead-in period where wear is built up. If a user suffers pain when they first introduce something to their shoe they are unlikely to continue wearing it.”

### 3.3. Human Volunteer Testing

Ten participants (seven male, three female) carried out the full protocol (mean age = 23 ± 4.2 years, mean body mass = 72 ± 7.3 kg, mean height 179 ± 9.3 cm). VAS scores were collected, collated, and compared between the *Control Condition* (Day 1, 3 h of walking with no insole fitted) and the *Insole Condition* (Day 4, 3 h of walking with insole fitted). In clinical terms, a difference in VAS scores of greater than or equal to 9.6 mm for the two conditions indicates a clinically-meaningful change in comfort level [11]. Table 8 shows the change in VAS score. The VAS scores obtained for the left and right insoles were averaged. In addition, paired *t*-tests were carried out to test for statistical significance between the VAS scores for overall foot, the heel, the mid-foot and the forefoot comfort (α = 0.05). 

**Table 8:** Comparison of mean (x) and standard deviation (σ) VAS scores for the control condition and the insole condition. Paired *t*-tests were used to test for statistical significance between the same time points for each condition.

Comfort Type	Left and Right VAS Average *Control Condition* (After 3 h)		Left and Right VAS Average *Insole Condition* (After 3 h)		Difference	Clinically Meaningful Difference According to Mills *et al*. [11]	*p*-Value
	x	σ	x	σ			
Overall Comfort	79.5	8.3	60.5	10.9	19	Yes	0.0002
Heel Comfort	76	9.2	59.5	6.6	16.5	Yes	0.0002
Midfoot Comfort	83.5	4.5	53.5	5.9	30	Yes	0.0001
Forefoot Comfort	87.5	4.6	73	6.1	14.5	Yes	0.0002

## 4. Changes to the Insoles Based on Results

Based on these findings, a human factors and comfort report was generated and disseminated to the insole designers, to provide them with the opportunity to address each problem the experts had identified in addition to the testimony from the experts and the data from the human volunteer testing. The report included high definition photographs of the identified problems and suggestions from experts as to how to address each identified problem (See Figure 7 for examples). Insole designers attempted to address each problem in turn based on priority. The experts who inspected the insoles were aware of the electronic circuitry, which was contained in the insoles and therefore took this into account when making recommendations, so as not to propose unattainable objectives for designers, who were seeking to not affect the durability and integrity of the insole.

**Figure 8:**
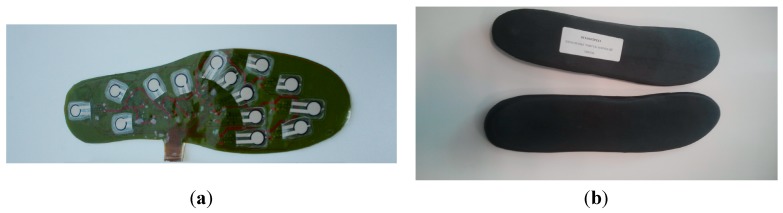
(**a**) The clear polyurethane layer was removed from the sensor layer to improve the flexibility of the insoles and (**b**) the prototype bottom layer (EVA) and top layer (EVA) respectively.

Table 9 summarizes the expert recommendations and the proposed design solutions. The changes outlined in Table 9 allowed designers to make the insoles both thinner (by at least 1 mm) and narrower (at least 3 mm) allowing them to fit in a wider range of shoes and reduce tightness for the wearer. Figure 8a shows the new thinner, more flexible sensor layer using the material Kapton, Figure 8b shows the bottom layer (EVA) and top layer (EVA) respectively.

**Table 9:** List of problems identified, recommendations for addressing them and how they were ultimately addressed.

Problem Number	Problem Identified	Priority Category	Expert Recommendations	How was the Problem Addressed in New Version?
1	The medial-longitudinal arch is too firm	High	Poron is a spongy shock absorbing material often used as a top cover for insoles, recommended that this or a similar spongy material such as EVA foam be used to alleviate the potential discomfort caused by the firm arch and by any other inconsistencies in the hardware layer of the insole	Introduction of softer EVA top layer which provided more cushioning and shock absorption than the leather
2	Lack of flexibility in the midfoot to rear foot region	Low	Review the materials that make up the middle layers and consider more flexible materials	Removal of the polyurethane encapsulation of the pressure sensors’ layer
3	Sensors are not flush with the surface of the insole	Low	Introduction of a softer top layer may negate the effect that protruding sensors have on the sole of the foot, Addressing of problem 1 may also solve this problem	Because the EVA layer is looser fitting than the leather layer, there is less chance of the sensors sticking out on the surface
4	Length and thickness of the insole will cause problems for manipulation and fitting	Low	The thickness of the insole needs to be reviewed as the current thickness was going to exclude too many types of shoes. Judging from the rigidity of the insoles it is clear that there users with dexterity problems will experience problems manipulating the insoles into certain types of shoes	Insole was less rigid and >1 mm thinner therefore manipulation into the shoe was easier
5	Pinch ridge around the outside of the insole causing problems for lateral movement and fit	Cosmetic	While it is clear that this exists due to nature of the encapsulation method being used, every effort should be made to reduce this so as to allow a better fit for the insole in the shoe. This ridge should be pared down to the minimum possible without affecting the integrity of the encapsulation	New insole slightly narrower with a smaller pinch ridge. A smaller pinch ridge was required because there was no need to bond (pinch) the leather layer
6	Lack of a proper heel cup	Cosmetic	While this may not be possible given the nature of the electronics, this heel should either be softened or shaped in some way to accommodate the contours of the foot	Unaddressed as introduction of Heel Cup would affect sensor output, the introduction of softer materials will afford more comfort for heel
7	Forefoot Rigidity	Cosmetic	See Problem 2 recommendations	Removal of polyurethane encapsulation material
8	Slippery Surface	Cosmetic	The introduction of Poron/EVA will prevent slippage. This population is susceptible to sores and irritation on the feet and any kind of movement of the foot against the insole was not recommended	EVA top layer has more grip and did not create a slippery interface with the foot

Like the first version of the insole, this version was exposed to numerous electrical and environmental assessments before being released for human factors analysis, such as waterproofing, electrical leakage/safety, sensor sensitivity and durability. This new version of the insole was now exposed to a second cycle of testing using the same use case as a guide to the context of use.

## 5. Results of Second Prototype (V2 Prototype) Test Cycle

The results of the human factors and Comfort Inspection and the subsequent human volunteer testing for the V2 Prototype of the insoles are presented in this section.

### 5.1. Human Factors and Comfort Inspection (V2 Prototype)

The same six experts used to inspect the V1 prototype also inspected the new V2 prototype of the insole using the same methodology. For this inspection, the experts were presented with the new prototypes and the list of the problem areas they identified in the V1 prototype. They were not made aware that the current devices were updated in response to the problems they identified and the recommendations they made. Experts were also not told what severity score they had originally assigned to each problem area. They were asked to assign severity scores to each of the original problem areas based on their inspection of the new prototype. Table 10 shows how the problems areas identified with the V1 prototype were severity rated for the V2 prototype.

**Table 10:** Problem severity ratings

Problem Number	How was the Problem Addressed?	V1 Prototype Severity Ratings	V2 Prototype Severity Ratings
1	Introduction of softer outer material, EVA layer which provides more cushioning and shock absorption than the leather	17	6
2	Removal of polyurethane layer and to increase flexibility	12	9
3	Because the EVA layer was not as tight as the leather layer, the sensors protruded less out on the surface	7	4
4	Insole was less rigid therefore manipulation into the shoe was easier	7	6
5	Insole was slightly thinner with a smaller pinch ridge. A smaller pinch ridge was required because there was no requirement to bond (pinch) the leather layer to the bottom EVA layer	6	4
6	The issue was not addressed as the introduction of a Heel Cup would affect sensor output, however some experts reduced the severity score for this problem by virtue of the softer materials used which afford more cushioning for the heel	5	4
7	Removal of polyurethane layer and introduction of middle EVA layer to increase flexibility	3	1
8	EVA had more grip and does not create a slippery interface with the foot	3	0

Of the eight problems, one was reduced from high priority to cosmetic, two were reduced from low priority to cosmetic and one was eliminated completely as a problem. Four maintained their original classification but received lower severity ratings. Experts were also asked to identify any new problems but no new problems were identified.

### 5.2. Expert Recommendations

Experts recommended carrying out the controlled protocol again with younger adults before moving onto further limited testing with older adults.

### 5.3. Human Volunteer Testing (V2 Prototype)

Ten participants (six male, four female) carried out the full protocol (mean age = 25 ± 9.3 years, mean body mass = 70 ± 7.2 kg, mean height 176 ± 4.6 cm). The same test and data analysis methodology was applied to the V2 prototype, comparing VAS scores for the *Control Condition* to VAS scores for the *Insole Condition* in order to establish if there was any significant comfort differences between the two conditions. Again we used Mills *et al*. [11] to check for clinical significance and paired *t*-tests to test for statistical significance. The results are summarized in Table 11.

**Table 11:** Comparison of mean (x) and standard deviation (σ) for 3 h VAS scores for *Control Condition* and *Insole Condition*. Paired *t*-tests were used to test for statistical significance between the same time points for each condition.

Comfort Type	Left and Right VAS Average *Control Condition* (after 3 h)		Left and Right VAS Average *Insole Condition* (after 3 h)		Difference	Clinically Meaningful Difference According to Mills *et al*. [11]	*p*-Value
	x	σ	x	σ			
Overall Comfort	81.5	11	77.5	8.4	4	No	0.39
Heel Comfort	80	10.3	83.5	14.2	3.5	No	0.41
Midfoot Comfort	82.5	13.33	72	12.7	10.5	Yes	0.14
Forefoot Comfort	81	8.6	80	17.4	1	No	0.49

## 6. Discussion

Through the use of a human factors and comfort assessment methodology we have guided the design of an instrumented insole, which at end of this cycle of development was suitable for testing with the target older adult end-user population. By using robust human factors testing techniques within an iterative process starting with a use case, we set the parameters, which informed the development of the initial V1 prototype. By engaging early in the process with all stakeholders and experts, we defined the context of use and user characteristics. The outcome of this process, led to the development of the V1 prototype, which was subsequently put through a rigorous comfort assessment. The comfort assessment revealed significant differences in the mean VAS scores recorded for the *Control Condition* and the *Insole Condition*. Expert-identified concerns expressed during the human factors and Comfort Inspection about the midfoot and heel region of the insoles were validated during the human volunteer testing phase. Participants complained about the firmness and rigidity of the insoles. The experts’ concerns presented in the human factors report highlighted, for the insole designers, the comfort and human factors problems present in the V1 prototype. It was made clear to designers that the current insole design rendered it unsuitable for testing with older adults.

**Figure 9:**
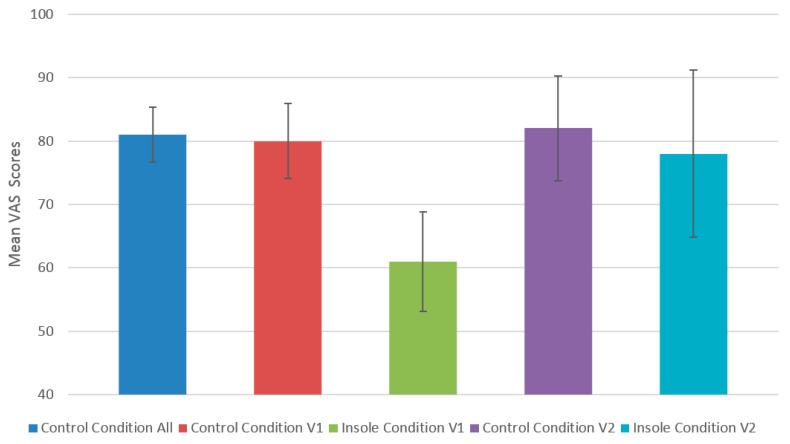
Comparison of VAS means after 3 h exposure to each condition. The first column shows the combined mean of both control conditions from the two human testing phases.

The V2 Prototype saw the leather top layer change to EVA layer. Furthermore, the insoles overall dimensions were reduced due to the removal of a polyurethane layer, which also improved flexibility. These changes to design resulted in significantly improved problem severity ratings in the expert inspection phase, with all of the problem severity ratings of the originally identified problems being reduced and half of the original problems being downgraded to a lower priority problem category. The reduction in severity ratings from the V1 prototype to the V2 prototype, during the human factors and comfort inspection, corresponded with improved VAS scores in the human volunteer testing of the V2 prototype. There was no statistically significant difference observed between VAS scores for the *Control Condition* and the *Insole Condition* for each area of the insole and as well as overall for the V2 prototype insole. In Figure 9 we compare VAS scores for the V1 prototype and the V2 prototype, as well as their respective controls.

When we consider the question “*Did we Meet or Exceed our Goal?*” a commonly asked question during a human-centered design process [27], we can be confident based on the data presented in Figure 9, that the latest version of the insole at a minimum has the same comfort characteristics of the user’s normal footwear. We feel that this was a realistic and valid goal to set [28]. However, due to the nature of the device and the safety advice received by the expert group, the device was not tested on the ultimate end-user for the technology, older adults. We consider this a limitation of the study.

The trade-off between providing a comfortable, wearable and safe insole against creating a functional, instrumented device was a challenge for the design team. The nature of the electronic components encapsulated within the insole can limit the flexibility, firmness, and dimensions of the insoles, while the use of certain materials could affect the sensitivity of the pressure sensors and the vulnerability of the electronic components to water damage. The trade-offs required, were made known to all stakeholders at an early stage of the project through the use case, thereby limiting the requirements for design changes further down the line. The experts who inspected the insole had an important role to play in recommending design changes, based on their assessment of the prototypes. Therefore the choice of experts is an important one and can result in either, informed and accurate design recommendations or in recommendations that do not guide the appropriate development of the design.

## 7. Conclusions

The human factors and comfort assessment methodology we have designed and implemented based on the principles of human-centered design (ISO 9241-210), has resulted in an instrumented insole design, which is now deemed suitable for exposure to older adults for clinical evaluation. Following the human-centered design process, we established a clear context of use though a use case and adopted the use of multi-disciplinary skills and perspectives and followed an iterative evaluation-driven process [29]. We have applied appropriate testing techniques given the context of use of the device being assessed [30]. This approach resulted in improved human factors and comfort scores for the device prototypes as they evolved during the design process and, thus, resulted in a better design.

## References

[1] Pappa I.P.I., Keller T., Mangold S., Popovic R., Dietz V., Morari M. (2004). A reliable gyroscope-based gait-phase detection sensor embedded in a shoe insole. IEEE Sens. J..

[2] Dyer P.S., Bamberg S.J.M. Instrumented insole *vs.* force plate: A comparison of centre of plantar pressure. Proceedings of the Annual International Conference of the IEEE Engineering in Medicine and Biology Society.

[3] US Food and Drug Administration (2011). Applying Human Factors and Usability Engineering to Optimize Medical Device Design.

[4] Armijo D., McDonnell C., Werner K. (2009). Electronic Health Record Usability: Evaluation and Use Case Framework.

[5] Harte R.P., Glynn L.G., Broderick B.J., Rodriguez-Molinero A., Baker P.M.A., McGuiness B., O’Sullivan L., Diaz M., Quinlan L.R., ÓLaighin G. (2014). Human centred design considerations for connected health devices for the older adult. J. Pers. Med..

[6] Van Netten J.J., Jannink M.J.A., Hijmans J.M., Geertzen J.H.B., Postema K. (2010). Use and usability of custom-made orthopaedic shoes. J. Rehabil. Res. Dev..

[7] Van Netten J.J., Jannink M.J.A., Hijmans J.M., Geertzen J.H.B., Postema K. (2010). Long-term use of custom-made orthopaedic shoes: 1.5-year follow-up study. J. Rehabil. Res. Dev..

[8] Nigg B.M., Khan A., Fisher V., Stefanyshyn D. (1998). Effect of shoe insert construction on foot and leg movement. Med. Sci. Sports Exerc..

[9] Miller J.E., Nigg B.M., Liu W., Stefanyshyn D.J., Nurse M.A. (2000). Influence of foot, leg and shoe characteristics on subjective comfort. Foot Ankle Int..

[10] Mündermann A., Nigg B.M., Stefanyshyn D.J., Humble R.N. (2002). Development of a reliable method to assess footwear comfort during running. Gait Posture.

[11] Mills K., Blanch P., Vicenzino B. (2010). Identifying clinically meaningful tools for measuring comfort perception of footwear. Med. Sci. Sports Exerc..

[12] International Organization for Standardization (2010). ISO 9241SO10:2010—Ergonomics of Human-system interaction—Part 210: Human-Centred Design for Interactive Systems.

[13] Thorleuchter D., Weck G., den Poel D.V. (2012). Usability Based Modelling for Advanced IT-Security—An Electronic Engineering Approach. Advances in Mechanical and Electronic Engineering.

[14] Van der Weegen S., Verwey R., Spreeuwenberg M., Tange H., van der Weijden T., de Witte L. (2013). The Development of a Mobile Monitoring and Feedback Tool to Stimulate Physical Activity of People with a Chronic Disease in Primary Care: A User-Centred Design. JMIR Mhealth Uhealth.

[15] Yue T., Briand L.C., Labiche Y. (2013). Facilitating the transition from use case models to analysis models: Approach and experiments. ACM Trans. Softw. Eng. Methodol..

[16] Jaspers M.W.M. (2009). A comparison of usability methods for testing interactive health technologies: Methodological aspects and empirical evidence. Int. J. Med. Inf..

[17] Fernandez A., Abrahão S., Insfran E. (2013). Empirical validation of a usability inspection method for model-driven Web development. J. Syst. Softw..

[18] Borycki E., Kushniruk A., Nohr C., Takeda H., Kuwata S., Carvalho C., Bainbridge M., Kannry J. (2013). Usability Methods for Ensuring Health Information Technology Safety: Evidence-Based Approaches. Contribution of the IMIA Working Group Health Informatics for Patient Safety. Yearb. Med. Inform..

[19] Manzari L., Trinidad-Christensen J. (2013). User-Centered Design of a Web Site for Library and Information Science Students: Heuristic Evaluation and Usability Testing. Inf. Technol. Libr..

[20] Sears A., Jacko J.A. (2009). Human-Computer Interaction: Development Process.

[21] Khajouei R., Peute L.W.P., Hasman A., Jaspers M.W.M. (2011). Classification and prioritization of usability problems using an augmented classification scheme. J. Biomed. Inform..

[22] Liu Y., Osvalder A.-L., Dahlman S. (2005). Exploring user background settings in cognitive walkthrough evaluation of medical prototype interfaces: A case study. Int. J. Ind. Ergon..

[23] Mahatody T., Sagar M., Kolski C. (2010). State of the Art on the Cognitive Walkthrough Method, Its Variants and Evolutions. Int. J. Hum. Comput. Interact..

[24] Zhang J., Johnson T.R., Patel V.L., Paige D.L., Kubose T. (2003). Using usability heuristics to evaluate patient safety of medical devices. J. Biomed. Inform..

[25] Rubin J., Chisnell D. (2008). Handbook of Usability Testing: How to Plan, Design, and Conduct Effective Tests.

[26] Eich E., Reeves J.L., Jaeger B., Graff-Radford S.B. (1985). Memory for pain: Relation between past and present pain intensity. Pain.

[27] Sauro J., Lewis J.R. (2012). Quantifying the User Experience: Practical Statistics for User Research.

[28] Rosenbaum S., Rohn J.A., Humburg J. A Toolkit for Strategic Usability: Results from Workshops, Panels, and Surveys. Proceedings of the SIGCHI Conference on Human Factors in Computing Systems.

[29] Giacomin J. (2014). What Is Human Centred Design?. J. Des..

[30] Stanton N.A., Salmon P.M. (2012). Human Factors Methods: A Practical Guide for Engineering and Design.

